# Redox-Mediated Regulation of Mitochondrial Biogenesis, Dynamics, and Respiratory Chain Assembly in Yeast and Human Cells

**DOI:** 10.3389/fcell.2021.720656

**Published:** 2021-09-07

**Authors:** Stefan Geldon, Erika Fernández-Vizarra, Kostas Tokatlidis

**Affiliations:** Institute of Molecular Cell and Systems Biology, College of Medical, Veterinary and Life Sciences, University of Glasgow, Glasgow, United Kingdom

**Keywords:** mitochondria, biogenesis, protein import, redox signaling, ROS, respiratory chain assembly, MIA

## Abstract

Mitochondria are double-membrane organelles that contain their own genome, the mitochondrial DNA (mtDNA), and reminiscent of its endosymbiotic origin. Mitochondria are responsible for cellular respiration via the function of the electron oxidative phosphorylation system (OXPHOS), located in the mitochondrial inner membrane and composed of the four electron transport chain (ETC) enzymes (complexes I-IV), and the ATP synthase (complex V). Even though the mtDNA encodes essential OXPHOS components, the large majority of the structural subunits and additional biogenetical factors (more than seventy proteins) are encoded in the nucleus and translated in the cytoplasm. To incorporate these proteins and the rest of the mitochondrial proteome, mitochondria have evolved varied, and sophisticated import machineries that specifically target proteins to the different compartments defined by the two membranes. The intermembrane space (IMS) contains a high number of cysteine-rich proteins, which are mostly imported via the MIA40 oxidative folding system, dependent on the reduction, and oxidation of key Cys residues. Several of these proteins are structural components or assembly factors necessary for the correct maturation and function of the ETC complexes. Interestingly, many of these proteins are involved in the metalation of the active redox centers of complex IV, the terminal oxidase of the mitochondrial ETC. Due to their function in oxygen reduction, mitochondria are the main generators of reactive oxygen species (ROS), on both sides of the inner membrane, i.e., in the matrix and the IMS. ROS generation is important due to their role as signaling molecules, but an excessive production is detrimental due to unwanted oxidation reactions that impact on the function of different types of biomolecules contained in mitochondria. Therefore, the maintenance of the redox balance in the IMS is essential for mitochondrial function. In this review, we will discuss the role that redox regulation plays in the maintenance of IMS homeostasis as well as how mitochondrial ROS generation may be a key regulatory factor for ETC biogenesis, especially for complex IV.

## Introduction

Mitochondrial biogenesis is essential for cell fitness and viability. Mitochondria are double membrane bound organelles composed of an outer membrane (OM), intermembrane space (IMS), inner membrane (IM), and matrix that harbors its own mitochondrial genome, the mtDNA. This mtDNA encodes only 13 polypeptides in humans or 8 polypeptides in yeast cells that are translated into key components of the respiratory chain complexes embedded in the IM. Assembly of the respiratory chain complexes relies on the protein import process through which most of these subunits find their way into the organelle.

A large number of proteins imported into the intermembrane space (IMS) are cysteine rich proteins that are imported via the mitochondrial import and assembly (MIA) pathway which involves oxidation of specific cysteine residues and subsequent folding of proteins into their mature conformation thereby trapping them in this compartment. As many of the substrates of this pathway include a variety of chaperones, proteases, mitochondrial dynamics factors and assembly factors for the respiratory chain complexes, and redox regulation of the IMS plays a crucial role in a wide variety of processes within the mitochondria. In this review we give a brief overview of the protein import pathways moving on to a more detailed presentation of the redox-regulated MIA pathway with a focus on the yeast and human systems. We then discuss the redox regulation features of mitochondria dynamics and proteases in the IMS following on with an analysis of the redox regulation mechanisms that are pertinent for the assembly process of the electron transport chain (ETC) complexes. Finally, we examine the possible role of reactive oxygen species (ROS) on maintaining the homeostasis of the respiratory chain.

## Overview of Mitochondrial Protein Import Pathways

**FIGURE 1:**
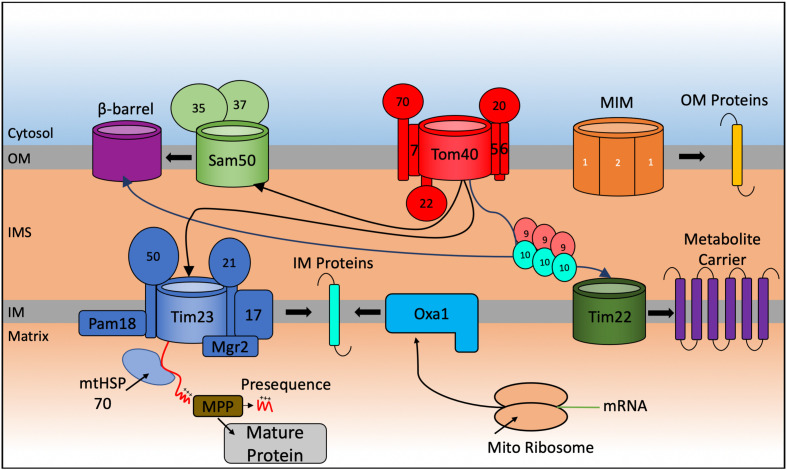
Schematic representation of the general import pathways for proteins destined for localization to the mitochondria. Incoming nuclear-encoded proteins destined for import into the mitochondria follow a number of particular import pathways depending on the sub compartment where they become localized. Matrix targeted proteins follow the presequence pathway, passing through the TOM40, and TIM23 translocases where they become cleaved by matrix processing proteins (MPP) in the matrix to form the mature protein. Preproteins targeted to the outer membrane (OM) are inserted by the Sam complex if they are β-barrel proteins and via the mitochondrial import machinery (MIM) if they are α-helical containing proteins. Integration of preproteins into the inner membrane (IM) involves interaction with the Tim9-Tim10 chaperone complex that targets proteins to the TIM22 translocase for membrane insertion or via a variation of the TIM23 complex that promotes integration of the protein into the IM. Mitochondrial encoded proteins are synthesized on mito ribosomes located in the matrix and inserted into the IM via the Oxa1 complex.

The mitochondrial proteome consists of 1,000–1,500 proteins (Sickmann et al., 2003; Reinders et al., 2006; Pagliarini et al., 2008; Rath et al., 2021) but only eight in the yeast *S. cerevisiae* and thirteen in humans, and other mammalian species, are encoded in the mitochondrial genome and translated inside the organelle. This implies that ∼99% of all mitochondrial proteins (1163 polypeptides in human mitochondria according to the last compendium of MitoCarta 3.0 (Rath et al., 2021) are nuclear encoded, translated on cytosolic ribosomes and guided to the mitochondria by a variety of cytosolic chaperones. From here they can be sorted within the organelle (Figure 1).

### Protein Entry Into Mitochondria

The translocase of the outer membrane (TOM) complex forms the entry gate for most imported mitochondrial proteins. The TOM complex is composed of seven different functional and receptor subunits. Precursors with a cleavable N-terminal presequence are recognized on the surface of mitochondria by the Tom20 receptor, while the Tom70 receptor recognizes precursor proteins that lack such a presequence but contain an internal targeting signal instead (Brix et al., 1997; Yamamoto et al., 2009; Backes et al., 2018). Tom22 is the central receptor that helps recruit both Tom20 and Tom70 to the TOM core complex and is involved in transferring preproteins from the receptor subunits to the translocation pore (Van Wilpe et al., 1999; Yamano et al., 2008). The structure of the *S. cerevisiae* TOM complex was recently solved using cryo-electron microscopy at a 3.8Å resolution (Araiso et al., 2019). This detailed structure revealed the architecture of the TOM entry gate providing a framework how the additional subunits Tom5, Tom6, Tom7 and Tom22 are organized within the complex. We will not provide a detailed analysis here as the TOM structural and functional features have been reviewed extensively (Shiota et al., 2015; Araiso et al., 2020).

### Protein Sorting in the Inner Mitochondrial Compartments

Following translocation through the TOM complex in the OM, proteins are sorted through different import pathways into the matrix, the IM and the IMS (Jensen and Johnson, 2001; Grevel et al., 2019; Hansen and Herrmann, 2019). Most of the precursor proteins targeted to the matrix are synthesized with an N-terminal positively charged cleavable presequence (Vögtle et al., 2009) and follow the matrix targeting pathway, which accounts for almost two thirds of all mitochondrial proteins. The presequence translocase of the inner membrane (TIM23 complex) is the key translocon that allows passage of these matrix-targeted precursor proteins through the inner membrane. The positively charged region of the presequence is recognized by the TOM receptors Tom20 and Tom22 that facilitate the translocation across the OM. The Tim50 subunit feeds the positively charged region into the Tim23 channel (Dayan et al., 2019). The translocation of these precursors through the pore of the TIM23 complex is dependent on the membrane potential (Δψ) across the inner membrane generated from the activity of the respiratory chain complexes. Additionally, the TIM23 complex is also involved in releasing proteins, containing a strongly hydrophobic transmembrane segment adjacent to the matrix-targeting presequence, laterally into the inner membrane. These proteins start to engage with the Tim23 channel guided by their presequence but become stalled because of their strong hydrophobic “stop-transfer” signal in the translocation pore of the Tim23 channel, preventing their complete import into the matrix. A specific cleavage of the ‘stop-transfer’ signal by the IMS localized IMP protease releases the mature protein into the IMS. The translocation-arrest mechanism of such bipartite presequence containing preproteins requires a specific conformation of the TIM23 complex that contains Tim17, Tim21, and Tim23, termed the TIM23^SORT^ translocase (Chacinska et al., 2010). By contrast, the TIM23 complex associated with the matrix-localized Pam18 (TIM23^PAM^) blocks the lateral release into the lipid bilayer and instead promotes import into the matrix (Schendzielorz et al., 2018). Protein transport into the matrix requires the ATP-dependent activity of the mitochondrial heat shock protein 70 (mtHsp70) (Pais et al., 2011) in addition to the membrane potential. Once in the matrix, the presequence is cleaved off from the precursor by the matrix processing peptidase (MPP), giving rise to the mature mitochondrial protein (Yang et al., 1991).

The mitochondrial metabolite inner membrane carrier proteins (at least 35 proteins in *S. cerevisiae* and more than 50 in mammalian cells) are critical for the trafficking of small metabolites and are synthesized without a cleavable presequence. They interact with the Tom70 receptor, engage with the TOM channel and are guided to the inner membrane TIM22 complex, by a number of small TIM chaperones in the IMS that escort them in the aqueous IMS preventing their aggregation. This import and insertion process constitutes the carrier pathway, and it is membrane potential-dependent but independent of ATP hydrolysis in the matrix (Sirrenberg et al., 1996).

### Protein Insertion Into the Mitochondrial Outer Membrane

The mitochondrial outer membrane contains either β-barrel membrane proteins (which are absent from the inner membrane) or α-helical transmembrane proteins. Precursors of the β-barrel proteins are first recognized by the TOM complex and then transferred to the sorting and assembly (SAM) complex from the IMS side of the OM. A recent cryo-electron microscopy structure of the SAM complex revealed that it is made up of two copies of the central subunit Sam50, forming a β-barrel channel (Takeda et al., 2021) that allows the lateral release of the precursor proteins into the outer membrane (Paschen et al., 2003; Klein et al., 2012). The SAM complex also contains the Sam35 and Sam37 proteins found on the cytosolic side of the outer membrane. Sam35 is thought to stabilize the precursor-Sam50 interaction, while Sam37 is involved in the release of substrate proteins from the SAM complex (Chan and Lithgow, 2008; Kutik et al., 2008). Transfer from the IMS to the OM is mediated by the IMS small TIM chaperones that transiently bind to exposed hydrophobic regions preventing their aggregation (Wenz et al., 2015; Weinhäupl et al., 2018). α-helical membrane proteins in the OM follow a dedicated pathway via the mitochondrial import machinery (MIM) complex (Becker et al., 2011; Dimmer et al., 2012).

### Import of Proteins Into the Intermembrane Space – The MIA Pathway

**FIGURE 2:**
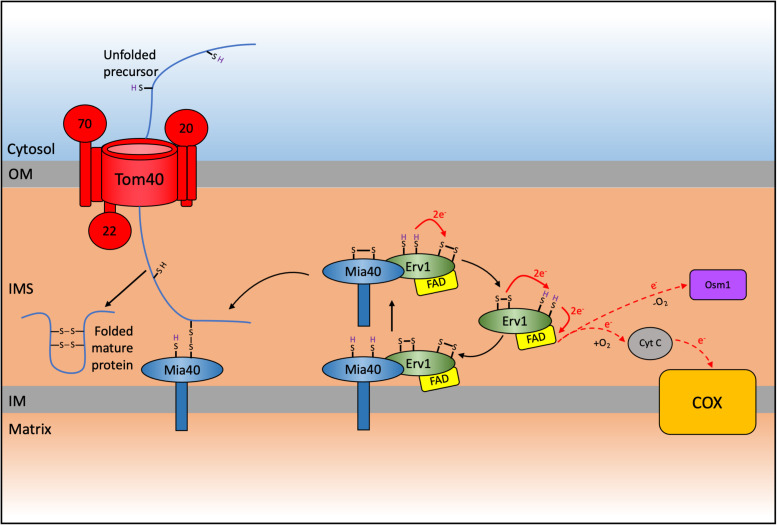
Schematic representation of the mitochondrial import and assembly (MIA) pathway in yeast. A subset of proteins destined for the intermembrane space (IMS) are imported via the MIA pathway which results in the oxidative folding of these substrates thereby trapping them in the IMS. Nuclear encoded CX_n_C motif containing proteins are first imported through the TOM complex. These proteins contain an IMS-Targeting signal (ITS) that interacts with Mia40 resulting in the oxidation and subsequent folding of the substrate protein, thereby trapping it in the IMS. This interaction leaves Mia40 in a reduced state that must be reoxidized to allow it to further oxidize incoming substrates. This reoxidation is facilitated by interaction with Erv1 with subsequent electron transfer to cytochrome *c* and complex IV (COX) of the respiratory chain in aerobic conditions, or onto Osm1 in anaerobic conditions. The system varies in humans, where CHCHD4/MIA40 is a soluble IMS protein that interacts with membrane bound AIFM1 and with ALR/GFER/ERV1 (Reinhardt et al., 2020).

A detailed analysis of the proteome of the mitochondrial IMS for the yeast *S. cerevisiae* revealed that around 50 proteins were localized to this sub-compartment (Vögtle et al., 2012). In humans, the IMS proteome accounts for about 5% of the total mitochondrial proteome (53 proteins) (Rath et al., 2021). The majority of these IMS-located proteins lack a typical mitochondrial targeting sequence and are instead characterized by conserved cysteine residues organized into twin CX_n_C (typically either CX_3_C or CX_9_C) motifs that are necessary for their import, correct folding and maturation. The oxidative folding or mitochondrial protein import and assembly (MIA) pathway relies on the function of Mia40, in yeast, and CHCHD4 (also known as MIA40), in humans, as the key protein that facilitates the disulfide bonds in these proteins (Mesecke et al., 2005; Figure 2).

Many of these classical MIA substrates are vital for the biogenesis and function of mitochondria as, for example, the members of the small Tim protein family, including Tim 8, Tim 9, Tim 10, and Tim13 (Sideris and Tokatlidis, 2007). These proteins form the Tim9-Tim10 and Tim8-Tim13 chaperone complexes that are essential to deliver hydrophobic proteins to the TIM22 complex for insertion into the inner membrane. Other examples of MIA substrates are proteins necessary for the biogenesis of the ETC complexes. These will be discussed in detail in subsequent sections and include proteins with a dual CX_9_C motif that are involved mostly in the assembly and maintenance of cytochrome *c* oxidase (COX), also known as complex IV (CIV) (Gabriel et al., 2007; Koch and Schmid, 2014a; Habich et al., 2019a; Gladyck et al., 2021). It is, however, clear that Mia40 can recognize proteins that do not have CX_n_C motifs, like the yeast Erv1 protein (with a CX_15_C motif) (Terziyska et al., 2007; Kallergi et al., 2012), yeast Atp23 (with 10 cysteine residues not organized in any specific cysteine motif (Weckbecker et al., 2012), yeast Mix23 (with an unusual twin cysteine CX_13_C/CX_14_C motif (Vögtle et al., 2012; Zöller et al., 2020), or human COA7 containing thirteen Cys residues (Mohanraj et al., 2019). Other proteins without classical CX_n_C motifs that have been described as substrates of CHCHD4/MIA40 also include p53, AK2 and MICU1 (Zhuang et al., 2013; Petrungaro et al., 2015). It is also of note that Ccs1, the copper chaperone for Cu,Zn superoxide dismutase (Sod1), is a Mia40 substrate in yeast but the IMS import of the human ortholog (CCS1) does not depend on CHCHD4/MIA40 (Groß et al., 2011; Suzuki et al., 2013).

The other main component of the yeast MIA pathway is the FAD-dependant sulfhydryl oxidase Erv1 (Chacinska et al., 2004; Mesecke et al., 2005). The human ortholog is known as ERV1, ALR, or GFER (Lange et al., 2001; Di Fonzo et al., 2009). Mia40 and Erv1 function as a disulfide relay system to catalyze the import of proteins into the IMS through an oxidative folding process. This results in introduction of disulfide bonds into the imported proteins, promoting their folding into their native conformation and thereby trapping them in the IMS. Substrates of the MIA pathway are translated cytosolically and pass through the TOM complex in a reduced and unfolded state (Lu et al., 2004; Sideris and Tokatlidis, 2007). During translocation these substrates interact with Mia40, which acts as a trans site receptor to drive protein import into the IMS (Milenkovic et al., 2007; Sideris and Tokatlidis, 2007; Peleh et al., 2016).

The yeast Mia40 has an N-terminal transmembrane domain that inserts the protein to the inner mitochondrial membrane and exposes its C-terminal domain to the IMS (Chacinska et al., 2004; Naoé et al., 2004; Terziyska et al., 2005). The human MIA40 (or CHCHD4) lacks this N-terminal domain and instead is found as a soluble protein of around 15 kDa in the mitochondrial IMS (Hofmann et al., 2005) where it interacts with the membrane anchored AIFM1 and this interaction facilitates the import of MIA40 into the IMS (Hangen et al., 2015). The C-terminal region was recently demonstrated to be important in maintaining the stability of the protein in the cytosol where it can often reside for extended periods as a consequence of slow import kinetics during AIFM1-mediated import (Murschall et al., 2020). However, all Mia40 homologs contain a highly conserved core domain of around 8 kDa that harbors six conserved cysteine residues that are folded into a coiled-helix-coiled-helix (CHCH) domain (Banci et al., 2009). The structure of the human and yeast homologs was solved by NMR and X-ray crystallography, respectively (Banci et al., 2009; Kawano et al., 2009). The invariant cysteines in this domain form a redox-active CPC motif that can readily switch between oxidized and reduced states, followed by a twin CX_9_C motif that forms two structural disulfide bonds. The CHCH domain folds to form a characteristic hydrophobic cleft where substrates can bind and interact with Mia40 (Banci et al., 2009; Sideris et al., 2009). Most Mia40 substrates contain a hydrophobic sequence, also known as an IMS-targeting signal (ITS, Sideris et al., 2009) or alternatively termed the mitochondrial IMS-sorting signal (MISS, Milenkovic et al., 2009). This is a 9 amino acid internal peptide sequence that is necessary and sufficient for IMS targeting of proteins to Mia40. Deletion of the ITS results in a complete loss of import (Sideris et al., 2009). The sequence aids the first interaction with Mia40 via hydrophobic stacking (Milenkovic et al., 2009; Sideris et al., 2009). This initial interaction orientates the substrate toward Mia40 to allow the formation of a transient intermolecular disulfide bond between the second cysteine of the redox active CPC motif of Mia40 and the docking cysteine of the ITS in the imported substrate (Banci et al., 2009; Koch and Schmid, 2014b). This transient disulfide bond is subject to a nucleophilic attack by another cysteine in the substrate to form an intramolecular disulfide bond. This promotes the release of Mia40 and the correct folding of the protein, thereby trapping it in the IMS and completing its import process.

The introduction of a disulfide into a substrate by Mia40 leaves it in a reduced state and unable to oxidize any further imported proteins. Thus, to maintain a functional MIA pathway, Mia40 needs to continually be re-oxidized by interaction with Erv1, which contains three cysteine pairs that are highly conserved (Mesecke et al., 2005). The FAD-binding catalytic core domain has a CXXC redox-active disulfide (Cys130 – Cys133) and a C-terminal CX_16_C structural disulfide (Cys156 and 179). A third disulfide bond (Cys30 – Cys33) is present within the flexible N-terminal of the protein, which is natively disordered and is involved in the interaction with reduced Mia40 and the transfer of electrons toward the redox active-site disulfide (Lionaki et al., 2010; Banci et al., 2011a, Banci et al., 2013). From here electrons are transferred to a final electron acceptor via the FAD cofactor (Banci et al., 2012). Under aerobic conditions Erv1 can either transfer electrons directly onto molecular oxygen which results in the production of hydrogen peroxide within the IMS, or onto cytochrome *c*, the mobile electron transfer protein that donates electrons to CIV (Allen et al., 2005; Dabir et al., 2007; Daithankar et al., 2009; Banci et al., 2012; Peker et al., 2021). Alternatively, under anaerobic conditions the fumarate reductase Osm1 can be used as an electron acceptor (Neal et al., 2017).

Intriguingly, Mia40 itself is a substrate of the MIA pathway requiring interactions with endogenous Mia40 during its own import, which occurs in three steps. First, Mia40 is inserted through the Tim23 translocon in the inner mitochondrial membrane. Next, the core domain of the protein is folded through interactions with endogenous Mia40. Lastly, Mia40 interacts with Erv1 to oxidize the CPC motif to produce functional Mia40 that can oxidize incoming substrates of the MIA pathway (Chatzi et al., 2013).

### The Small Tim Chaperones

The small Tim proteins that function as chaperones in the IMS were the first discovered substrates of the MIA pathway, interacting with Mia40 to control their redox-regulated import (Chacinska et al., 2004). These small Tims possess non-cleavable internal targeting signals (ITS) that harbor conserved cysteine motifs to target them to the mitochondria (Milenkovic et al., 2009; Sideris et al., 2009). The cysteines are arranged in a CX_3_C motif, a classical substrate motif for Mia40 (Koehler, 2004). The ITS signal directs the small Tims to the IMS where they are imported by the MIA pathway through direct interaction with Mia40 (Chacinska et al., 2004; Sideris et al., 2009). This interaction results in the oxidation of the small Tims, leading to the formation of intramolecular disulfide bonds and trapping the proteins in the IMS.

The small Tims form hexameric protein complexes consisting of either Tim9 – Tim10 or Tim8 – Tim13 with three subunits of each Tim protein. Structural analysis of the complexes revealed the subunits take on the form of an α-propellor with two helical blades that radiate from a narrow central pore (Webb et al., 2006; Beverly et al., 2008). The subunits are stabilized by the formation of intramolecular and structural disulfide bonds. Within the IMS these complexes act as chaperones to transport incoming hydrophobic precursors across this compartment to the TIM22 complex for their insertion into the IMM. The helper of Tims 13 (Hot13p) was also shown to be involved in maintaining the small Tims in their active conformation (Curran et al., 2004).

The presence of the redox-regulated disulfide machinery in the IMS raises the question of a broader level of redox regulation in this sub-compartment that may affect not just the MIA pathway itself or import substrates for this pathway, but other important proteins and process linked to the IMS. In the next sub-sections, we will discuss in particular the redox regulation of mitochondrial dynamics and the effect of redox control on the IMS protease system. Both of these are critical for many functions of mitochondria and highlight the broader ramifications of mitochondria-specific redox control pathways.

### Redox Regulation of Mitochondrial Dynamics

Mitochondria undergo coordinated processes of fission and fusion, known as mitochondrial dynamics, to regulate their size, shape, and integrity. There are several key proteins involved in the control of mitochondrial dynamics that have been demonstrated to be redox-regulated. Mitofusin proteins (Mfn1/Mfn2) are large GTPases that are implicated in mitochondrial fusion. This process has been shown to be triggered by oxidative stress, characterized by an accumulation of oxidized glutathione (GSSG), which in turn leads to the assembly of higher order Mfn oligomers mediated by the formation of disulfide bonds between conserved cysteine residues in the C-terminal region (Shutt et al., 2012). This region has been recently identified to be topologically located in the IMS and the conserved Cys demonstrated to be redox sensitive (Mattie et al., 2018). Redox regulation in this compartment mediates the formation of disulfide bonds in the Mfn proteins thereby driving oligomerization and triggering mitochondrial fusion. Therefore, the redox state of the IMS is likely to play a role in controlling mitochondrial dynamics.

ROS modulator 1 (ROMO1) was identified as a key regulator of mitochondrial fusion, with knockout cells displaying increased fragmented mitochondrial networks. ROMO1 is also involved in the generation of ROS by complex III (CIII) of the ETC and can form disulfide bridges to become incorporated into inactive high-molecular weight complexes (Chung et al., 2006; Norton et al., 2014). Disulfide formation occurs between the Cys15 and Cys79 residues in response to oxidative stress, inhibiting fusion and maintaining a fragmented mitochondrial network (Norton et al., 2014). More recently ROMO1 was found to be required for the import of the human inner membrane YME1L protease (Richter et al., 2019).

During mitochondrial fusion there is separate merging of the outer and inner mitochondrial membranes. Fusion of inner mitochondrial membranes involves heterotypic interactions between the dynamin-like GTPase optic atrophy 1 (OPA1) and cardiolipin (Ban et al., 2017; Liu and Chan, 2017). Mammalian OPA1 is an inner membrane associated protein that exists in either a long form (L-OPA1) or soluble short-form (S-OPA1) conformation (Del Dotto et al., 2018). Conversion of OPA1 between these two isoforms occurs as a result of proteolytic cleavage of L-OPA1 that is catalyzed by the OMA1 and YME1L proteases (Anand et al., 2014). A balance of both the long- and short- isoforms of OPA1 depends on these proteases and is required to maintain normal mitochondrial morphology.

### Redox Regulation of Proteases in the IMS

YME1L, the human ortholog of yeast Yme1, is an integral membrane protein with its C-terminal domain exposed to the intermembrane (Stiburek et al., 2012). Yme1 is a subunit of the inner membrane anchored oligomeric complex known as the i-AAA protease that displays both ATP-dependent proteolytic and chaperone-like activities in the IMS (Leonhard et al., 1996, 1999; Schreiner et al., 2012). Yme1 is involved in the degradation of misfolded inner membrane proteins and more recently shown to be implicated in the clearance of the small Tim9 and Tim10 IMS chaperones (Leonhard et al., 2000; Baker et al., 2012). YME1L is rapidly degraded by OMA1, an ATP-independent protease, upon oxidative stress conditions (Rainbolt et al., 2015). This suggests that YME1L is a stress-sensitive protease and thus degradation of YME1L under oxidative stress conditions may provide a mechanism to segregate damaged mitochondria from the healthy pool, by restricting the ability of YME1L to regulate the fusion activity of OPA1 through its degradation. More recently it was demonstrated that YME1L adopts unique conformations depending on the ATP and ROS concentrations (Brambley et al., 2019). Under oxidative stress conditions, YME1L undergoes a conformational change, exposing OMA1 recognition sites that were previously unreachable in basal conditions, further supporting the mechanism of YME1L degradation by OMA1 during oxidative stress (Brambley et al., 2019).

Oma1, responsible for mediating OPA1 degradation, is an IMS facing ATP-independent metalloprotease embedded in the inner mitochondrial membrane displaying enhanced activity in response to stress conditions (Käser et al., 2003; Head et al., 2009). Oma1 is synthesized as a pre-pro-protein of 60 kDa that upon import into the mitochondria is proteolytically processed to a mature 40 kDa form (Head et al., 2009). The protein is normally dormant under normal physiological conditions but becomes rapidly activated upon changes in membrane potential, chronic hyperpolarization and oxidative stress (Bohovych et al., 2014). The 40 kDa Oma1 is proposed to be a stress-sensitive pro-protein that undergoes further autocatalytic cleavage in response to stress insults to produce the active form of the protein that is involved in proteolysis of substrates (Baker et al., 2014).

Oma1 exists in a semi-oxidative state in both yeast and mammalian cell types and the activity and stability of the homo-oligomeric complex was found to be altered in a redox sensitive manner. Two evolutionary conserved cysteine residues, Cys272 and Cys332, which are exposed to the IMS can form a disulfide bond that likely influences the stability of the oligomeric complex. It is proposed that the formation of this disulfide is controlled by the redox status of the cysteine residues which act as a redox sensor to influence the proteolytic activity of Oma1 in response to deviations from normal cell homeostasis (Bohovych et al., 2019).

A subset of IMS-located proteases has been shown to be involved in the proteolytic control of ETC assembly and stability, including the m-AAA, i-AAA and Oma1. These enzymes control the proteolysis of unassembled, misfolded OXPHOS subunits to prevent the formation of dysfunctional ETC subunits (Bohovych et al., 2015). Oma1 in particular is involved in the degradation of non-hemylated cytochrome *c* oxidase subunit 1 (Cox1) (Bestwick et al., 2010b; Khalimonchuk et al., 2012). In human mitochondria, the proteolytic activity of YME1L was shown to be involved in preventing the accumulation of non-assembled respiratory chain subunits including COX4, MT-ND1 and NDUFB6 (Stiburek et al., 2012).

In *S. Cerevisiae*, Atp23 was identified as an IMS protease required for the processing of the mtDNA-encoded subunit 6 of the mitochondrial ATPase (Atp6) (Osman et al., 2007). Atp23 contains ten cysteine residues, that during the import and folding into mitochondria form five disulfide bonds. The reduced Atp23 precursor is translocated into the IMS where Mia40 is then involved in introducing the disulfide bonds to promote folding of Atp23 into its mature conformation (Weckbecker et al., 2012).

The morphology of the inner membrane is regulated by the mitochondrial contact site and cristae organization system (MICOS) complex (Harner et al., 2011). Mic19, a peripheral inner membrane component of this complex is involved in the maintenance of the architechture of the inner membrane. Human MIC19 has five cysteine residues, four of which are arranged in a twin CX_9_C motif in the coiled-coil helix coiled-coil helix fomain, a typical motif for Mia40 substrates. This domain was shown to be essential for import of MIC19 into mitochondria and MIC19 demonstrated to form a transient disulfide-bond intermediate involving its Cys193 residue (Darshi et al., 2011, Darshi et al., 2012). Yeast Mic19, however, lacks the typical CX_9_C motif, and instead contains two conserved cys residues in a CX_10_C motif. Yeast Mic19 was identified in two different redox states in the mitochondria. The oxidized form displays an intermolecular disulfide with Mic60, which is not essential for its integration into the complex, but does play a role in the stability of the MICOS complex (Sakowska et al., 2015). This suggests that Mic19 may be a redox-sensor regulator of MICOS function and, therefore, of maintenance of inner membrane morphology.

## Redox-Regulation of OXPHOS Components and Biogenesis Factors

Many of the import, dynamics and redox-regulated processes mentioned so far culminate in the biogenesis of the oxidative phosphorylation (OXPHOS) system. This is physically located in the mitochondrial inner membrane (therefore in contact to the IMS on the one side) and it is composed of the four complexes (complexes I-IV) of the respiratory or ETC, which transfer reducing equivalents from NADH or FADH_2_ to oxygen, reducing it to water, using the two mobile electron carriers: coenzyme Q (CoQ) and cytochrome *c*. Electron transfer through complexes I, III and IV is coupled with proton pumping from the matrix to the IMS. This electrochemical gradient generates the protonmotive force (pmf) employed by the ATP synthase (or complex V) for the synthesis of the majority of the cellular ATP in aerobic eukaryotes (Wikström et al., 2015). Complexes I, III, IV, and V are large multimeric enzymes whose structures span the IMM, with subunits that protrude either in the matrix, the IMS or both (Letts and Sazanov, 2017). The processes of assembly and maturation of these complexes are intricate and require the action of a significant number of proteins, generically called assembly factors, that are not part of the mature complex but are necessary for ETC biogenesis. The structure and assembly of the respiratory complexes seem to be conserved for the most part between yeast and humans, except for the absence of a multimeric, and proton pumping complex I in *S. cerevisiae* (Signes and Fernandez-Vizarra, 2018). There is an additional level of complexity in the organization of the ETC, which is the formation of supercomplex structures. In yeast, all of CIV is associated with the invariantly dimeric CIII (CIII_2_) in III_2_IV_1__–__2_ stoichiometries (Hartley et al., 2019; Rathore et al., 2019). In mammalian mitochondria, CI, CIII_2_ and CIV associate with different stoichiometries into supercomplexes, in which CIII_2_ is always present, that coexist with the individual non-associated complexes (Letts and Sazanov, 2017; Lobo-Jarne and Ugalde, 2018).

Several structural subunits and assembly factors directly involved in OXPHOS biogenesis are Cys-containing proteins located in the IMS and, therefore, potentially subject to redox regulation (Habich et al., 2019b; Reinhardt et al., 2020). In addition, many of these are MIA40/CHCHD4 substrates (Table 1).

**TABLE 1:** IMS-located Cys-rich structural subunits of the ETC complexes and assembly factors.

Name	Molecular role	Target complex	Contains twin Cx_9_C motifs?	MIA40/CHCHD4 substrate?	Pathological variants identified to date?	References
NDUFA8	Structural	CI	Yes	Yes	Yes	Szklarczyk R. et al., 2011; Fischer et al., 2013; Zhu et al., 2016; Tort et al., 2020; Yatsuka et al., 2020
NDUFB7	Structural	CI	Yes	Yes	No	Szklarczyk et al., 2012; Zhu et al., 2016
NDUFB10	Structural	CI	No	Yes	Yes	Zhu et al., 2016; Friederich et al., 2017; Helman et al., 2021
NDUFS5	Structural	CI	Yes	Yes	No	Szklarczyk et al., 2012; Zhu et al., 2016
NDUFAF8?	Assembly	CI	Yes	Not known	Yes	Floyd et al., 2016; Alston et al., 2020
C9orf72	Assembly	CI	No	Yes	Yes	Wang et al., 2021
UQCRH/Qcr6	Structural	CIII	Yes	Yes	No	Vögtle et al., 2012
COX6B*/Cox12	Structural	CIV	Yes	Yes	Yes	Massa et al., 2008; Szklarczyk et al., 2012; Abdulhag et al., 2015
CMC1	Assembly	CIV	Yes	Yes	No	Horn et al., 2008; Bode et al., 2015; Bourens and Barrientos, 2017a
CMC2	Assembly	CIV	Yes	Yes	No	Horn et al., 2008; Longen et al., 2009
COA4/CMC3/CHCHD8	Assembly	CIV	Yes	Yes	No	Longen et al., 2009; Bestwick et al., 2010a; Bode et al., 2013; Fischer et al., 2013
COA5/Pet191	Assembly	CIV	Yes	Yes?	Yes	Khalimonchuk et al., 2008; Huigsloot et al., 2011
COA6/C1orf31	Assembly	CIV	Yes	Yes	Yes	Baertling et al., 2015; Stroud et al., 2015; Ghosh et al., 2016; Maghool et al., 2020; Pacheu-Grau et al., 2020
COA7	Assembly	CIV	No	Yes	Yes	Kozjak-Pavlovic et al., 2014; Lyons et al., 2016; Higuchi et al., 2018; Mohanraj et al., 2019
COX11	Assembly	CIV	No	No	No	Tzagoloff et al., 1993; Hiser et al., 2000; Carr et al., 2002; Banci et al., 2004
COX17	Assembly	CIV	Yes	Yes	No	Moira Glerum et al., 1996a; Amaravadi et al., 1997; Beers et al., 1997; Horng et al., 2004; Banci et al., 2008a; Oswald et al., 2009
COX19	Assembly	CIV	Yes	Yes	No	Nobrega et al., 2002; Rigby et al., 2007; Bode et al., 2015
CHCHD7/Cox23	Assembly	CIV	Yes	Yes	No	Barros et al., 2004; Banci et al., 2012; Dela Cruz et al., 2016
SCO1	Assembly	CIV	No	No	Yes	Moira Glerum et al., 1996b; Valnot et al., 2000; Leary et al., 2004, 2007, 2009
SCO2	Assembly	CIV	No	No	Yes	Moira Glerum et al., 1996b; Papadopoulou et al., 1999; Jaksch et al., 2000; Leary et al., 2004, 2007, 2009

*Two isoforms of COX6B exist: the ubiquitously expressed COX6B1 and the testis specific COX6B2 (Sinkler et al., 2017).

The Cys residues in these proteins are important for their biogenesis (i.e., their import and stability in the IMS), but in some cases also for their function (Gladyck et al., 2021). Most members of this group of proteins are also interesting from a medical point of view, as pathological variants in several genes encoding them have been found associated with respiratory chain deficiency and mitochondrial disease (Fernandez-Vizarra and Zeviani, 2021). Interestingly, the majority of IMS-located assembly factors appear to be involved in the maturation of CIV (Khalimonchuk and Winge, 2008; Longen et al., 2009; Bourens et al., 2013; Timón-Gómez et al., 2018; Gladyck et al., 2021). In addition to these, COA8 is a particular case of a CIV assembly factor, located in the IM facing the matrix, containing Cys residues within and immediately after its MTS and whose import and/or stability appears to be redox regulated (Signes et al., 2019).

### Role of IMS Proteins in Complex I Biogenesis

The MIA pathway has been thoroughly studied using the yeast *S. cerevisiae* as a reference model (Chatzi and Tokatlidis, 2013). However, there is no multimeric complex I (CI) present in this unicellular organism, having instead three monomeric NADH dehydrogenases denominated Ndi1, Nde1, and Nde2 (Velázquez and Pardo, 2001). Nonetheless, the strong dependence of the mammalian CI biogenesis on the MIA pathway was made evident by the fact that Apoptosis Inducing Factor Mitochondria Associated 1 (AIFM1) deficiency, which was discovered to be an important interactor of CHCHD4 in the IMS (Hangen et al., 2015). AIFM1 was originally discovered as a caspase-independent effector of cell death when migrating from the mitochondrion to the nucleus (Susin et al., 1999). However, it soon became clear that AIFM1 defects were related with a strong mitochondrial CI deficiency (Vahsen et al., 2004; Bénit et al., 2008). Subsequent studies revealed that loss of AIFM1 function produced a more generalized combined respiratory chain deficiency in mouse and human cells (Ghezzi et al., 2010; Delavallée et al., 2020), but the full ramifications of the AIFM1-CHCHD4 interaction remain to be resolved (Reinhardt et al., 2020). More recently and using *Drosophila melanogaster* models, it was suggested that the dependency of CI biogenesis on AIFM1 was not direct but a consequence of decreased Mic19 levels instead (Murari et al., 2020).

**FIGURE 3:**
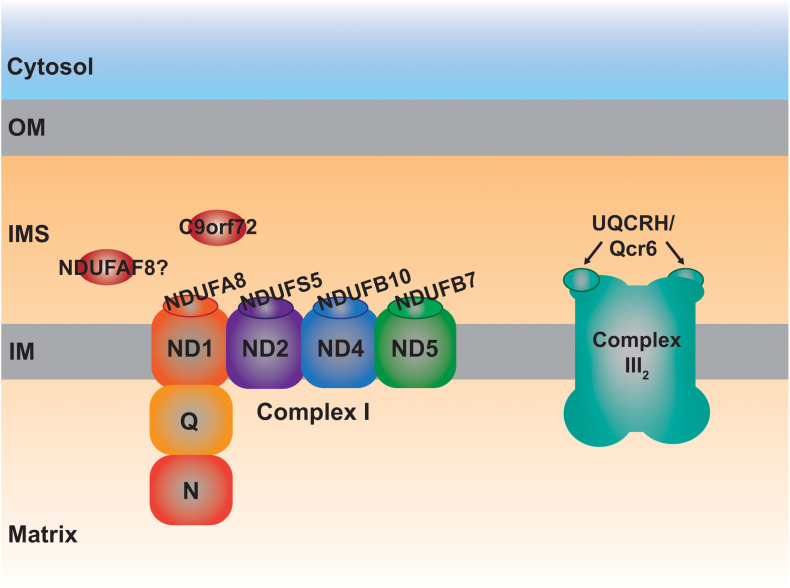
Schematic representation of the MIA40 protein substrates located in the IMS involved in the assembly of complexes I and III_2_. Each one of the IMS CI structural subunits, NDUFA8, NDUFS5, NDUFB10, and NDUFB7 belongs to a different module (ND1, ND2, ND4, and ND5, respectively) of the membrane arm, responsible for proton pumping. The peripheral arm of CI is composed of the quinone binding (Q) and NADH dehydrogenase (N) functional modules. UQCRH, Qcr6 in yeast, is an IMS-located supernumerary subunit.

More specifically, four CI supernumerary subunits, i.e., outside the fourteen evolutionarily conserved core subunits, are located in the IMS (Zhu et al., 2016). These are **NDUFA8**, **NDUFB7**, **NDUFS5** and **NDUFB10** (Table 1 and Figure 3), the first three subunits are CHCHD4 substrates containing typical twin Cx_9_C motifs (Szklarczyk D. et al., 2011; Fischer et al., 2013). NDUFB10 is also imported into the IMS via CHCHD4 (Friederich et al., 2017), although it appears to contain two extra disulfide bonds, all of which most probably contribute to the stability of the complex (Zhu et al., 2016). In fact, lack of any of these four subunits is detrimental to the assembly of CI (Stroud et al., 2016). In accordance, a pathological variant in a conserved Cys residue in NDUFB10 and those in *NDUFA8* predicting changes in conserved Arg residues located at the vicinity of the CX_9_C motifs result in the structural loss of CI (Friederich et al., 2017; Tort et al., 2020; Yatsuka et al., 2020), reinforcing the importance of these domains for the import and/or stability of these IMS subunits and, consequently, that of the whole complex.

**NDUFAF8** is a CI assembly factor that was identified through proteomic studies as an interactor of other CI-related proteins and then determined to be encoded by a mitochondrial disease gene (Floyd et al., 2016; Alston et al., 2020). Sequence analysis predicted its IMS localization due to the presence of twin CX_9_C motifs. However, even though the exact submitochondrial localization has not been proven experimentally, it is likely that NDUFAF8 is located in the matrix due to its strong interaction with NDUFAF5, a well-known matrix protein (Floyd et al., 2016; Alston et al., 2020).

Defects in **C9orf72** have been associated with the development of amyotrophic lateral sclerosis and frontotemporal dementia (ALS/FTD) but the molecular functions of this protein are still unclear (Braems et al., 2020). Very recently it was determined that a proportion of C9orf72 is imported into the mitochondrial IMS via AIFM1/CHCHD4 where it binds to the prohibitin complex and the m-AAA protease (Merkwirth and Langer, 2009) regulating the proteolysis of the CI assembly factor TIMMDC1 (Guarani et al., 2014) and, consequently, the amounts of fully assembled CI (Wang et al., 2021).

### Role of IMS Proteins in Complex III Biogenesis

**Qcr6** in yeast and **UQCRH** in mammals, is the only structural component of the ten subunits that constitute the cytochrome *bc*_1_ complex (or complex III; CIII_2_) localized completely in the IMS and containing Cx_9_C motifs (Iwata et al., 1998; Hunte et al., 2000; Szklarczyk R. et al., 2011; Vögtle et al., 2012). Qcr6/UQCRH is a supernumerary subunit, not involved in the catalytic Q-cycle, whose function is still unclear. The first observations in Δ*qcr6* strains showed normal growth in non-fermentable carbon sources and CIII_2_ activity. However, further investigations revealed a temperature-sensitive *petite* phenotype associated with a defect in the assembly of the *bc*_1_ complex and lack of maturation of cytochrome *c*_1_ (Cyt1), one of the three catalytical subunits (Yang and Trumpowers, 1994). Using different deletion strains, including that of Qcr6, it was determined that the subunit was incorporated on its own in the middle states of the assembly pathway (Zara et al., 2009). However, proteomic studies using a human cell line with a strong defect in CIII_2_ assembly, revealed the formation of intermediates containing UQCR10, cytochrome *c*_1_ (CYC1) and most probably UQCRH as well (Protasoni et al., 2020). One can speculate that Qcr6/UQCRH would somehow contribute to the maturation of CYC1 before it is incorporated into nascent CIII_2_ also in human mitochondria. This possible function and the involvement of a redox regulatory mechanism through the Cys residues present in UQCRH, remains an interesting aspect to investigate.

### Role of IMS Proteins in Complex IV Biogenesis

**FIGURE 4:**
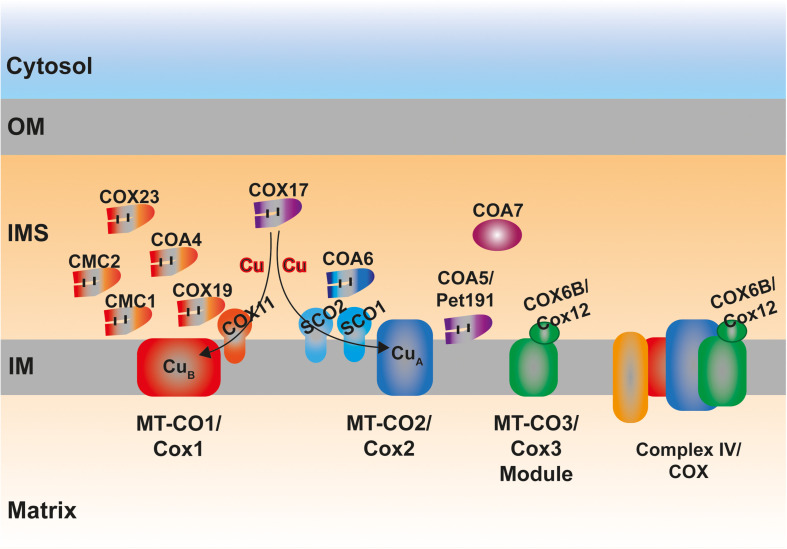
Schematic representation of the MIA40 protein substrates located in the IMS involved in the assembly of complex IV (COX). The proteins involved in the stabilization and metalation of MT-CO1 and MT-CO2 are indicated in red and blue color, respectively. COX17 is the archetypal twin CX_9_C protein, mediating copper (Cu) delivery to the active centers of both MT-CO1 and MT-CO2. The exact molecular role of COA5/Pet191 and COA7 is still unknown. COX6B, Cox12 in yeast, is the only COX subunit fully localized in the IMS.

Of all the ETC complexes, cytochrome c oxidase (COX) or complex IV (CIV) is probably the most heavily dependent on Cys-rich redox regulated IMS proteins for its biogenesis and function. COX catalysis, oxidizing cytochrome *c*, and reducing molecular oxygen, involves the presence of two heme *a* moieties and one copper (Cu_B_ center) ion in Cox1/MT-CO1, and two Cu ions (Cu_A_ center) in Cox2/MT-CO2 (Wikström et al., 2018). The processes of metal cofactor insertion, i.e. hemylation and Cu delivery, occur in the IMS and therefore, a significant number of Cys-rich proteins located in this compartment seem to be involved in these processes, especially in the formation of the Cu_A_ site, as well as in the stabilization of these two COX subunits in early assembly stages (Khalimonchuk and Winge, 2008; Jett and Leary, 2018; Timón-Gómez et al., 2018; Gladyck et al., 2021; Table 1 and Figure 4). In addition to all the assembly factors that will be described below, there is one structural subunit exclusively localized in the IMS, which is yeast **Cox12** or human **COX6B1** (ubiquitous isoform) or **COX6B2** (testis-specific isoform), containing one CX_9_C and one CX_10_C motif (Tsukihara et al., 1996). The first mammalian CIV structure was in dimeric form, appearing COX6B1 to serve as a bridge and stabilize the COX dimer, as well as modulating the interaction with cytochrome *c* (Kadenbach and Hüttemann, 2015). However, it was recently determined that the monomer harboring COX6B1, could also be isolated and that it displayed high activity (Shinzawa-Itoh et al., 2019). Apart from a stabilization role, a function in Cu delivery to the active center of Cox2/MT-CO2 was also proposed for Cox12/COX6B1 (Ghosh et al., 2016). In any case, this structural subunit is clearly important for completion of the assembly process and for the activity of the enzyme, as mutations destabilizing COX6B1 or Cox12 result in assembly defects and COX deficiency in human and yeast (Massa et al., 2008; Abdulhag et al., 2015).

**Cmc1** was firstly identified in yeast as an IMS-located protein containing twin CX_9_C motifs and imported by the MIA pathway that was necessary for COX expression (Horn et al., 2008; Bourens et al., 2012; Fischer et al., 2013). Later, it was determined that human CMC1 was necessary for the stabilization of MT-CO1, being released from the first intermediate of the MT-CO1 module once this subunit joins the early nuclear-encoded subunits COX4 and COX5A (Bourens and Barrientos, 2017a). The exact function of CMC1 is still not clear but it was proposed to either bind Cu itself or to somehow assist the process of MT-CO1 metalation (Horn et al., 2008; Bourens and Barrientos, 2017a).

**Cmc2** was identified in yeast via a screening of all the genes encoding for CX_9_C motif-containing proteins (Longen et al., 2009). Deletion of Cmc2 produced a respiratory-deficient phenotype associated with low COX activity (Longen et al., 2009; Horn et al., 2010). Cmc2 is homologous to and interacts with Cmc1, cooperating in COX assembly with a different non-overlapping molecular role. The human and *C. elegans* CMC2 orthologs contain the conserved Cys residues, are localized in mitochondria and appear to be involved in CIV biogenesis as well (Horn et al., 2010).

**Cmc3** is another member the group of Cx_9_C proteins identified in yeast, also related with COX biogenesis (Longen et al., 2009). This protein was later renamed **Coa4** (cytochrome c oxidase assembly number 4) and its involvement in CIV assembly was confirmed being identified as a genetic suppressor of mutations in Shy1 modeling those found in the human ortholog SURF1 and associated with Leigh syndrome (Petruzzella et al., 1998; Zhu et al., 1998; Bestwick et al., 2010b). The twin CX_9_C motives in Coa4/Cmc3 are highly conserved in the human protein, which is known also as **CHCHD8**, being also imported into the IMS using the oxidative folding pathway (Fischer et al., 2013), although its direct relationship with human CIV biogenesis has not been proven experimentally yet.

**Pet191** was also determined to be important for COX activity in a screen for respiration deficient yeast strains (McEwen et al., 1993). It contains a variation of the twin Cx_9_C motifs (i.e., two Cx_9_C and one Cx_10_C) and there are still conflicting evidences to whether its import in the IMS is mediated by Mia40 and Erv1 or not (Khalimonchuk and Winge, 2008; Bragoszewski et al., 2013). Although its involvement in COX biogenesis was clearly proven, Pet191 is not a Cu-binding protein (Khalimonchuk and Winge, 2008). **C2orf64**, also known as **COA5**, is the human ortholog of *S. cerevisiae* Pet191 (Szklarczyk et al., 2012). A pathological variant in C2orf64 was associated with COX activity and assembly defects. Although the exact molecular role of this protein remains unknown, its levels are increased in cells where the MT-CO2 copper chaperones SCO1 and SCO2 are defective (Leary et al., 2013). In addition, the pattern of CIV subassemblies accumulated in the patient-derived cultured cells indicated a function in the early steps of assembly (Huigsloot et al., 2011).

**Coa6** in yeast is a COX assembly factor as well as an IMS-localized protein and a substrate of the MIA import pathway (Vögtle et al., 2012). The human and zebrafish orthologs also contain the conserved CX_9_CX_n_CX_10_C motifs and have been proven to be important for the correct biogenesis of CIV (Szklarczyk et al., 2012; Ghosh et al., 2014; Baertling et al., 2015; Pacheu-Grau et al., 2015; Stroud et al., 2015). The fact that Cu supplementation restored the growth of defective strains in fermentative substrates, the fact that COA6 binds Cu *in vitro*, together with the connection of the protein with SCO1 and SCO2 and with MT-CO2 maturation, led to the idea that COA6 was a Cu-binding protein involved in the process of delivery of this metal to the Cu_A_ site (Ghosh et al., 2014, 2016; Baertling et al., 2015; Pacheu-Grau et al., 2015; Stroud et al., 2015; Bourens and Barrientos, 2017b; Aich et al., 2018). However, more recent studies have suggested that COA6 is a thiol-reductase binding SCO1 and/or SCO2 and catalyzing the reduction of critical Cys residues in SCO1 and/or SCO2 as well as MT-CO2 allowing the transfer and binding of Cu to MT-CO2 (Soma et al., 2019; Pacheu-Grau et al., 2020). The exact COA6 binding partners and mechanisms are still not completely clear as the interpretation of the results varied in different laboratories depending on the experimental approach used (Maghool et al., 2020).

**COA7/C1orf63/SELRC1** is a protein only present in metazoa that was originally studied as a Mitofilin interactor whose decreased levels were associated with defects in the respiratory chain (Kozjak-Pavlovic et al., 2014). Although one report localized this protein within the mitochondrial matrix (Lyons et al., 2016), more detailed biochemical analyses determined its IMS localization (Kozjak-Pavlovic et al., 2014; Mohanraj et al., 2019). Although it does not contain the typical Cx_3_C or Cx_9_C motifs, it is a Cys-rich protein that it is imported inside the mitochondria via CHCHD4 (Mohanraj et al., 2019). The first indication that COA7 is involved in COX biogenesis came from the fact that pathological variants in the COA7 gene are associated with mitochondrial CIV deficiency in humans (Lyons et al., 2016; Higuchi et al., 2018; Ban et al., 2021). Recent data suggest that it is a haem-binding protein involved in the early stages of COX assembly (Formosa et al., 2021). The right Cys composition in COA7 was shown to be crucial for IMS import efficiency and for its stability (Mohanraj et al., 2019).

**Cox11** deficiency in yeast results in decreased COX levels (Tzagoloff et al., 1990). Originally proposed to be involved in heme *a* synthesis (Tzagoloff et al., 1993) it is clear now that it is a Cu binding protein involved in the formation of the Cu_B_ center in Cox1, through conserved Cys residues in its Cu-binding motif (Hiser et al., 2000; Carr et al., 2002; Banci et al., 2004). A gene encoding a protein showing high homology to yeast Cox11 is present in the human genome (Petruzzella et al., 1998) and although the direct involvement of COX11 in metalation human CIV assembly has not yet been proven, it was shown to interact with CIV subunits and assembly factors (Vidoni et al., 2017).

**Cox17** is the archetypal twin CX_9_C IMS protein involved in Cu binding in mitochondria, and it has been extensively studied at the functional and structural levels both in yeast and human systems (Gladyck et al., 2021). Cox17 plays a fundamental role in COX biogenesis by donating Cu to Sco1 for metalation of Cox2 on one side, and to Cox11 for the formation of the Cu_B_ center, on the other (Horng et al., 2004; Cobine et al., 2006, 2021). The highly conserved residues contained in the Cx_9_C motifs and in the Cu binding motif are necessary for import using the MIA system, binding of Cu, oligomerization and promoting CIV assembly (Heaton et al., 2000, 2001; Palumaa et al., 2004; Mesecke et al., 2005; Banci et al., 2008a, b, 2011b). Although Cox17 is found both in the IMS and the cytosol and for that reason it was proposed to act as a Cu shuttle to the inside of mitochondria (Beers et al., 1997), other investigations point out to the fact that the only active partially oxidized form of Cox17 is the one present in the IMS (Maxfield et al., 2004; Palumaa et al., 2004; Banci et al., 2008b). COX17 is also an essential protein in mammals as knocking-out the mouse gene leads to embryonic lethality (Takahashi et al., 2002). Human cell lines with knock-down expression of COX17 display decreased amounts of MT-CO1 and MT-CO2 together with the accumulation of assembly intermediates containing MT-CO1 but lacking MT-CO2 (Oswald et al., 2009; Vanišová et al., 2019). Curiously, the proportion of CIV associated with complexes I and III within the supercomplexes was more sensitive to the loss of COX17 that the “free” complex (Oswald et al., 2009). In addition, intramitochondrial Cu amounts were reduced in human cells where COX17 expression was stably knocked down (Vanišová et al., 2019). All in all, these observations point out to a completely conserved function of COX17 between yeast and humans.

The amino acid sequence of **Cox19** is homologous to that of Cox17, containing the twin CX_9_C motifs but lacking the additional Cys residues of the Cu binding domain (Gladyck et al., 2021). As indicated by its name it is also a necessary factor for COX biogenesis (Nobrega et al., 2002). Similarly, the yeast and human proteins also localize both in the cytosol and mitochondria, being the steady-state levels and distribution of COX19 Cu-dependent (Nobrega et al., 2002; Leary et al., 2013). The import into the IMS is mediated by Mia40/CHCHD4 (Fischer et al., 2013) and Cox19 also shows Cu binding properties *in vitro* (Rigby et al., 2007), whereas, *in vivo* Cu binding by Cox19 appears to be less likely. COX19 interacts with COX11 in a redox regulated manner, most likely to keep the Cu-binding Cys residues of COX11 reduced and, therefore, in an active form (Bode et al., 2015; Cobine et al., 2021).

**Cox23** was again identified in *S. cerevisiae* as a protein necessary for COX biogenesis showing high homology to Cox17 (Barros et al., 2004) with a potential function in Cox1 maturation (Dela Cruz et al., 2016). Structural analysis of the human homolog CHCHD7/COX23, predicted the presence of an ITS and the dependence on MIA40 for its folding creating the typical disulfide bridges between the twin CX_9_C motifs (Banci et al., 2012). Interestingly, the levels of human COX23 decrease when the Cu binding proteins SCO1 and SCO2 are defective or in the presence of a Cu chelating agent (Leary et al., 2013).

**SCO1** and **SCO2** are not members of the CHCHD family, but they are IM tethered proteins facing the IMS containing Cys residues important for Cu binding, essential for their function in the metalation of MT-CO2 (Nittis et al., 2001; Leary et al., 2004). As pointed out above, there is clearly an interplay between SCO1 and SCO2 and the twin CX_9_C motif-containing chaperones involved in Cu delivery to the COX active sites, and this is illustrated by the fact that yeast Sco1 and Sco2 were originally identified as suppressors of Cox17 deficiency (Moira Glerum et al., 1996b). However, contrary to Sco1, the lack of Sco2 did not affect COX (Moira Glerum et al., 1996b). This differs from human mitochondria, where defects in both SCO1 and SCO2 result in CIV deficiency and pathological mutations in both these genes are associated with manifestations of mitochondrial diseases (Papadopoulou et al., 1999; Jaksch et al., 2000; Valnot et al., 2000; Leary et al., 2004; Fernandez-Vizarra and Zeviani, 2021). In human cells, SCO1 and SCO2 have non-overlapping cooperative roles in MT-CO2 maturation, where SCO2 acts upstream of SCO1 oxidizing its Cu-binding Cys residues during the process of Cu delivery to MT-CO2 (Leary et al., 2004, Leary et al., 2009). The importance of these processes not only for COX biogenesis but for other aspects of cellular health, is pointed out by the fact that functional SCO1 and SCO2 are required to maintain Cu homeostasis (Leary et al., 2007; Cobine et al., 2021).

## Reactive Oxygen Species-Regulated Assembly and Turnover of the ETC Complexes

**FIGURE 5:**
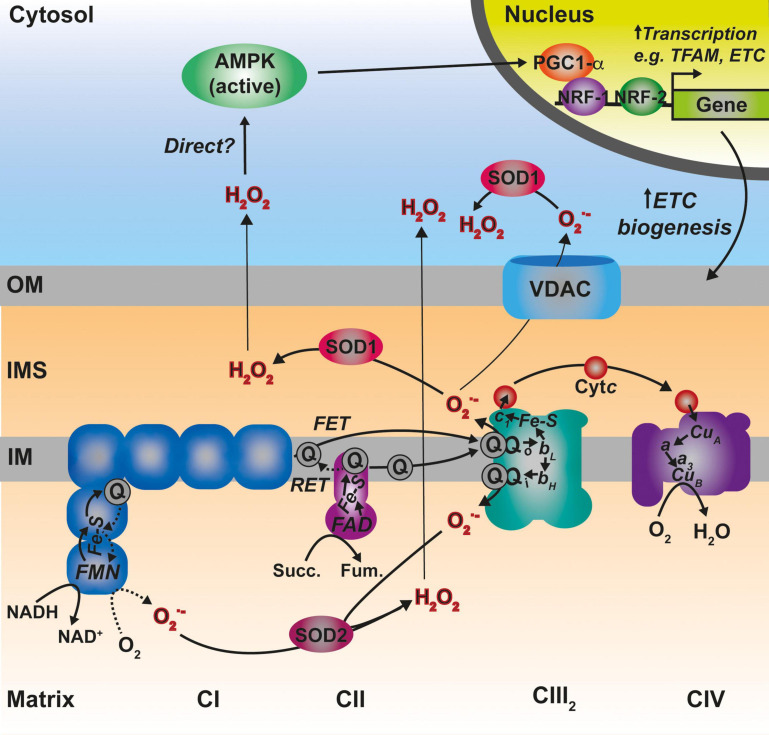
Main sites of ROS generation by the ETC and possible pathways of ROS-mediated regulation of mitochondrial biogenesis. Complex I (CI) represented in blue, generates superoxide (O_2_^–^) both via forward electron transfer (FET) or reverse electron transfer (RET) from a highly reduced coenzyme Q (Q) pool and high membrane potential. Two other prominent sites of O_2_^–^ production are the two Q binding sites in CIII2 (Qo and Qi). O_2_^–^ is dismutated to hydrogen peroxide (H_2_O_2_) by the two differently localized superoxide dismutases SOD1 and SOD2. See main text for details.

The production of ROS is inherent to the aerobic metabolic routes located in mitochondria (Murphy, 2009; Brand, 2016). These ROS are mainly superoxide (O_2_^–^), the majority of which is produced at the levels of complexes I and III (Brand, 2010, 2016), which is subsequently dismutated to hydrogen peroxide (H_2_O_2_), and the highly reactive and unstable hydroxyl radical (OH), produced through the Fenton reaction (Lambert and Brand, 2009). Mitochondria also produce other redox active compounds such as reactive nitrogen species (RNS), mainly nitric oxide (NO) (Ghafourifar and Cadenas, 2005), or H_2_S (Kimura et al., 2010; Paul et al., 2021), which induce modifications of Cys in proteins, modulating their activities (Habich et al., 2019b). However, discussion of these species is beyond the scope of this review, which focuses on ROS directly produced by the ETC (Figure 5), due to electrons slipping off of the system and escaping from the redox active centers to partially reduce oxygen. The idea that ROS are only harmful molecules leading to detrimental effects for the cell and causing different diseases and aging, is now changing (Scialò et al., 2017; Ji and Yeo, 2021). Nowadays, ROS are recognized as signaling molecules mediating a myriad of cellular responses (Holmström and Finkel, 2014; Reczek and Chandel, 2015). However, in parallel, cells have had to develop antioxidant defense systems to protect themselves from ROS overaccumulation and oxidative damage, whilst these antioxidant systems themselves might be involved in the ROS-mediated signaling as well (Holmström and Finkel, 2014; Reczek and Chandel, 2015). Consequently, a correct balance between ROS production and scavenging is important to ensure the appropriate cellular responses while preventing oxidative stress (Ji and Yeo, 2021). In fact, a moderate production of ROS is the basis of the phenomenon called “mitohormesis” leading to a stress adaptive response and ultimately to improved mitochondrial function and life extension (Schulz et al., 2007; Palmeira et al., 2019). In addition, it is becoming evident that ROS produced in the mitochondria play an important role in mediating mitochondrial biogenesis to compensate for ETC dysfunction (Dogan et al., 2018).

Superoxide radicals produced by CIII_2_ and glycerol-3-phosphate dehydrogenase (GPDH) are generated in both sides of the IM (the IMS and the matrix), whereas, all the other sites produce ROS in the matrix (Brand, 2010; Figure 5). In addition, ALR (Erv1) seems to be a significant contributor of O_2_^–^ radicals within the IMS (Daithankar et al., 2012). O_2_^–^ produced in the matrix side is dismutated to H_2_O_2_ by the mitochondrial Mn superoxide dismutase (SOD2) and can then diffuse to the cytosol (Boveris et al., 2006; Murphy, 2009), whereas, that released into the IMS can be transported through voltage-dependent ion channels into the cytosol (Han et al., 2003; Muller et al., 2004). Once in there, the Cu,Zn superoxide dismutase (SOD1) converts it to H_2_O_2_. SOD1 is dually localized in the cytosol and the IMS in mammals and yeast, and the IMS pool appears to be inactive in basal conditions and activated by modulating its redox state (Iñarrea et al., 2005, 2007). H_2_O_2_ in the cytosol is then able to modify the redox state of key Cys residues in target proteins, modulating the function of factors such as signaling regulatory kinases and phosphatases or transcription factors (Holmström and Finkel, 2014; Reczek and Chandel, 2015).

There are three aspects in which ROS production in mitochondria may influence directly the biogenesis and function of the ETC complexes: (1) by generating oxidation in the IMS and inducing changes in the redox state of key Cys residues and thus, in the structure and function of the redox-sensitive biogenesis factors; (2) the role of ROS produced inside mitochondria as signaling molecules to regulate ETC biogenesis; and (3) ROS-mediated protease activation inducing the degradation of components of the ETC complexes.

### Redox Homeostasis in the IMS and Regulation of ETC Biogenesis

Given the essentiality of the Cys-containing subunits and assembly factors of the ETC complexes, a correct redox homeostasis in the IMS is crucial for the biogenesis of the respiratory complexes. Thus, it is important to maintain the redox sensitive residues in the correct oxidation state for import and retention in the IMS, as well as for protein function (Habich et al., 2019a; Dickson-Murray et al., 2021). Considering the number of redox regulated IMS-located factors involved, this is particularly relevant for the assembly and metalation of CIV (Khalimonchuk and Winge, 2008; Jett and Leary, 2018). Although there are still many open questions as to how the IMS maintains its redox homeostasis (Dickson-Murray et al., 2021), it is clear that it contains several systems that ensure it, having glutathione as a central component (Calabrese et al., 2017). One such mechanism for maintaining homeostasis in this compartment is the retro-translocation of reduced proteins back into the cytosol through the Tom40 import pore protein. This provides a means to regulate the abundance of IMS proteins either in response to various changes in cellular physiology or for the removal of misfolded proteins by presenting them to protein quality control machineries located in the cytosol (Bragoszewski et al., 2015). Surprisingly, the IMS is not as oxidizing as one would expect, being the glutathione pools as highly reduced in yeast and human mitochondria as in the cytosol (Kojer et al., 2012; Fischer et al., 2013). In such a reducing environment, the essential oxidative folding pathway is maintained by limiting glutaredoxin (Grx) activity in the IMS (Kojer et al., 2015). As mentioned before, the IMS, where most of the Cys-rich redox sensitive proteins reside, receives significant amounts of ROS. H_2_O_2_ oxidizes target thiols of the Cys residues to form disulfide bonds, but also to higher oxidation states such as sulfenic, sulfinic and sulfonic acids, changing the functional properties of the Cys-containing proteins (Habich et al., 2019b). Sulfonylation is irreversible and sulfinylation was thought to be as well until an enzyme called sulfiredoxin (Srx) was identified in yeast and humans, catalyzing the reduction of Cys sulfinic acids *in vivo* (Jacob et al., 2004). Enzymes aimed to detoxify H_2_O_2_ and/or remove thiol modifications have been found in the IMS of both yeast and human mitochondria (Vögtle et al., 2012; Hung et al., 2014). Peroxiredoxins 3 and 4 (PRDX3 and 4) react directly with H_2_O_2_, reducing it to water, resulting in the oxidation of Cys residues that can be reduced again by thioredoxin 1 (TRX1), TRX1 is then recycled by Thioredoxin reductase (TRR) (Cardenas-Rodriguez and Tokatlidis, 2017; Habich et al., 2019b). In addition, glutathione peroxidase 3 (Gpx3) is found in the IMS, where it becomes actively targeted to through the addition of an N-terminal extension of 18 amino acids (N18) and is thought to play a role in H_2_O_2_ detoxification (Kritsiligkou et al., 2017). Translation of this long form of Gpx3 with the N18 extension can be modulated by H_2_O_2_ stress (Gerashchenko et al., 2012). The cytoplasmic isoform of GPX4, the human homolog of Gpx3, has also been found in mitochondria (Liang et al., 2009; Tadokoro et al., 2020) but the role it plays there is still unclear.

### ROS-Mediated Signaling Promoting ETC Biogenesis

**FIGURE 6:**
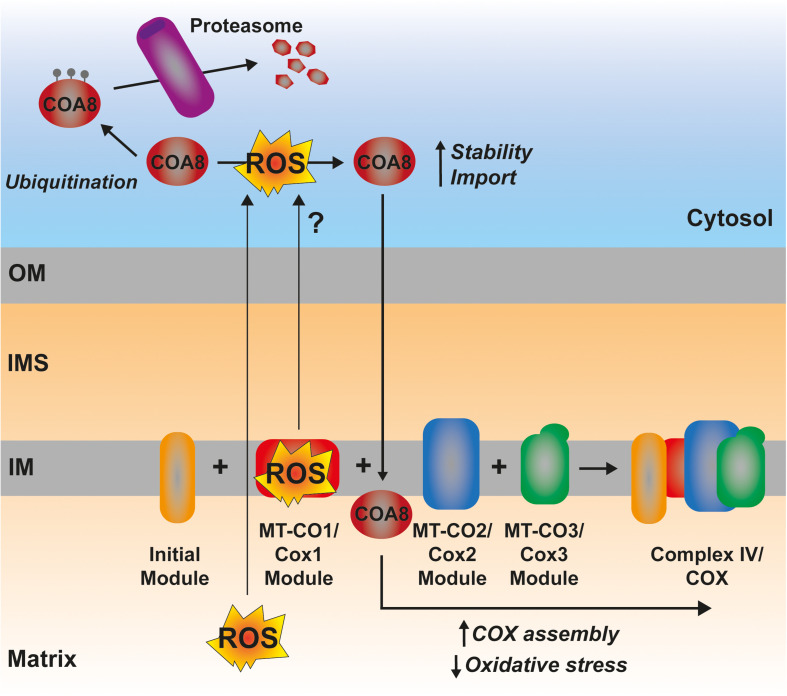
Possible mechanism of ROS-mediated CIV assembly regulation by COA8. The ROS produced by a hemylated partially assembled MT-CO1 could represent a signal triggering the increased import and/or stabilization of COA8, promoting CIV assembly and, thereby, reducing oxidative stress.

In certain situations, dysfunction or inhibition of CI, CIII, or CIV leads to increased ROS production (Verkaart et al., 2007; Kowaltowski et al., 2009; Brand, 2016; Dogan et al., 2018). However, as briefly discussed above, the nature and topology of these ROS is different depending on what complex is affected. In addition, the physio-pathological consequences can also diverge. CI can generate ROS both by “forward electron transfer (FET)” or by “reverse electron transfer (RET),” i.e., by transferring electrons from a highly reduced CoQ pool back to CI, in conditions where the membrane potential is high (Robb et al., 2018; Figure 5). This is relevant because ROS generated at the level of CI, but only if its via RET, are able to induce an adaptive program leading to improved mitochondrial activity and increased life span in *D. melanogaster* (Scialò et al., 2016). O2^–^ generated at the level of the first CoQ binding site of cIII_2_ (Q_*o*_), closest to the IMS, has been described to be the origin of signaling pathways such as the hypoxic responses (Guzy et al., 2005; Bell et al., 2007). At the mitochondrial level, the ROS-mediated hypoxic transcriptional program leads to the expression of specific COX isoforms, regulating the activity of the complex under these conditions (Bourens et al., 2013). Also, ROS produced in the mitochondria activate AMPK, which activates PGC-1α, a transcriptional master regulator of mitochondrial biogenesis (Jäer et al., 2007; Rabinovitch et al., 2017; Figure 5). This ROS-mediated signaling axis is necessary to induce an increase in mitochondrial mass to counteract CIV deficiency in muscle (Dogan et al., 2018). Apart from these mitochondrial remodeling effects mediated by transcriptional changes, an attractive possibility is that ROS produced in the mitochondria can directly modulate the activity of biogenetical factors such as COA8 (Figure 6). The absence of COA8 (formerly known as APOPT1) leads to CIV deficiency and the accumulation of MT-CO1-containing partially assembled species (Melchionda et al., 2014; Signes and Fernandez-Vizarra, 2018). COA8 was shown to be readily degraded by the proteasome in the cytosol but stabilized inside mitochondria when cells were stressed with both exogenous H_2_O_2_ or with mitochondrially targeted paraquat (mitoPQ), which induces the production of O_2_^–^ in the matrix (Signes and Fernandez-Vizarra, 2018). The COA8 sequence contains Cys residues within the mitochondrial targeting sequence that could potentially be oxidized by H_2_O_2_ in the cytosol, inducing its stabilization and/or mitochondrial import (Signes and Fernandez-Vizarra, 2018). Yeast strains deleted of Sco1 or Cox11 show increased sensitivity to acute hydrogen peroxide stress (Khalimonchuk et al., 2007; Veniamin et al., 2011). This effect was linked to impaired COX assembly leading to the accumulation of hemylated Cox1 intermediates acting as pro-oxidant species (Khalimonchuk et al., 2007). Interestingly, *COA8-null* patient derived fibroblasts produce more ROS when they are oxidatively challenged (Melchionda et al., 2014) and *dCoa8* knock-down flies are more sensitive to oxidative stress (Brischigliaro et al., 2019). A hypothesis that can be derived from these observations is that ROS produced because of the accumulation of pro-oxidant MT-CO1 subcomplexes serve as a signal to induce the assembly of CIV mediated by COA8, which also appeared to have a role in protecting MT-CO1 from oxidative stress induced degradation (Signes and Fernandez-Vizarra, 2018). More experimental data must be collected to confirm or disprove these ideas and to determine whether there are more redox sensitive assembly factors for CIV and the rest of the complexes, which may be regulated in this way to promote a rapid biogenetical response to regulate ETC function.

### ROS-Mediated Degradation of ETC Components

**FIGURE 7:**
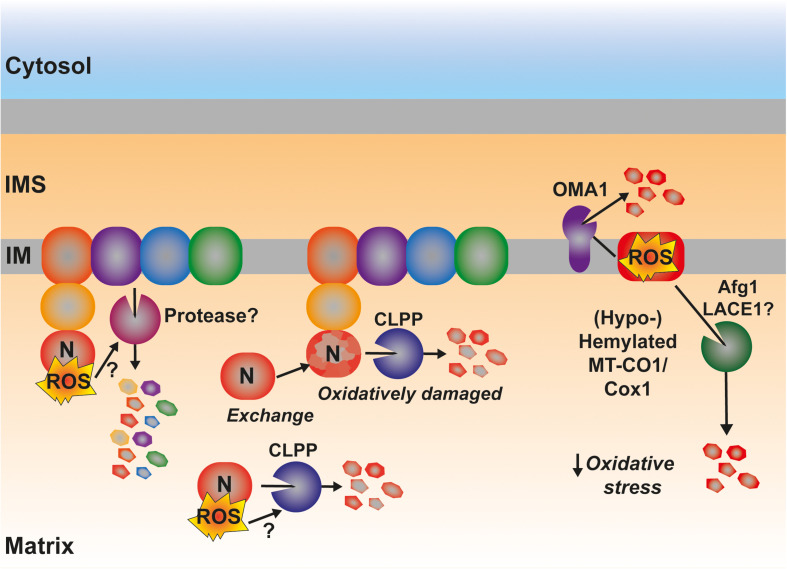
Possible involvement of ROS in the regulation of the degradation by active proteolysis of some of the catalytical components of the ETC. ROS produced by RET at the level of CI have been proposed to trigger the degradation of the full complex. The mechanistic details are still unknown. The matrix protease CLPP is responsible for the degradation of the catalytic N-module of CI, preventing its accumulation as an assembly intermediate in the mitochondrial matrix as well as promoting the interchange of an oxidatively damaged N-module for a newly synthesized one. Some Cox1 (MT-CO1) intermediates are actively degraded by the inner membrane-bound protease OMA1, which is redox activated. Other pro-oxidant Cox1 intermediates are degraded through a mechanism mediated by the AAA-ATPase Afg1 in yeast being LACE1 its human ortholog. See main text for details.

A much less explored yet interesting component in the control of the amounts and function of the ETC is the turnover, i.e., balance between synthesis and degradation, of its components (Szczepanowska and Trifunovic, 2021). In yeast, the accumulation of pro-oxidant COX subcomplexes is attenuated by the activity of the AAA-ATPase Afg1, facilitating the degradation of Cox1, Cox2, and Cox3 (Khalimonchuk et al., 2007; Figure 7). Afg1 human ortholog is LACE1, and it was shown to be involved in the degradation of nuclear-encoded CIV subunits (Cesnekova et al., 2016). As mentioned earlier, Cox1 accumulated due to the absence of the assembly factor Coa2 is degraded by Oma1 (Khalimonchuk et al., 2012) and Oma1 is redox regulated both in yeast and human mitochondria (Bohovych et al., 2019). Also in human mitochondria, in the case of defective CIV assembly at different stages with the accumulation of MT-CO1 module subassemblies, is associated with an increased turnover of the mtDNA-encoded CIV subunits (Leary et al., 2009; Bourens et al., 2014; Bourens and Barrientos, 2017a, b; Signes and Fernandez-Vizarra, 2018). Another ETC component in mammals, whose turnover is potentially regulated by ROS is CI (Figure 7). The assembly of this huge complex happens in a modular fashion and the N-module containing the redox active Fe-S clusters and flavin (FMN) is preassembled and then added in the last step of assembly (Guerrero-Castillo et al., 2017). CLPP is responsible for the turnover of both the pre-assembled and assembled N-module (Szczepanowska et al., 2020). In this case, one could think that this might be a way of preventing the accumulation of a highly reactive pro-oxidant species and to remove oxidatively damaged protein components in proximity to the N-module FMN center, which is one of the main sites of ROS production in mitochondria (Brand, 2016; Hirst and Roessler, 2016). Then again, mammalian CI shows a high interdependence with the other components of the ETC. Strong CIII_2_ and CIV assembly defects produce a secondary decline in CI amounts (Diaz et al., 2006; Protasoni et al., 2020; Čunátová et al., 2021). This was explained by the existence of an active degradation of fully assembled CI in response to RET-mediated ROS production and oxidative damage (Guarás et al., 2016). However, the exact mechanism and the protease/s mediating this accelerated turnover have not been defined yet, and other levels of regulation, i.e., repression of mitochondrial translation and attenuated assembly of the N-module, have been shown (Protasoni et al., 2020; Čunátová et al., 2021). In the case of the CIII_2_ defects originated by mutations in the central subunit MT-CYB, mitochondrial translation was not affected (Protasoni et al., 2020; Tropeano et al., 2020). However, the steady-state levels of CI subunits and specially those of the N-module were severely reduced (Protasoni et al., 2020; Páleníková et al., 2021). One can imagine that if the N-module is being preassembled but not incorporated into fully assembled CI, it will need to be degraded before its assembly, as previously shown (Szczepanowska et al., 2020). One can hypothesize that this might be ROS mediated and induced by a pro-oxidant intermediate, because xeno-expression of an alternative oxidase (AOX), which decreases ROS production by the ETC (Dogan et al., 2018; Robb et al., 2018; Szibor et al., 2020), resulted in increased amounts of N-module subunits and fully assembled CI in *MT-CYB* mutated human cells (Protasoni et al., 2020).

Whether the enhanced proteolysis of the redox active, and potentially damaging, CI and CIV subassemblies is directly modulated by ROS, and which are quality control proteases that are involved and regulated in this way, constitute extremely interesting topics to explore in the future.

## Conclusion and Perspectives

Mitochondria contain dedicated import and sorting machineries to direct mitochondrial proteins that are nuclear encoded and synthesized in the cytoplasm. The MIA pathway makes use of reduction and oxidation reactions involving particular Cys residues so that the correctly folded and functional proteins are retained in the IMS. Therefore, redox homeostasis in the IMS plays a crucial role in the regulation of the ETC complexes because essential factors involved in their maturation are proteins containing twin Cx_9_C motifs and substrates of Mia40/CHCHD4. In order to guarantee a correct ETC biogenesis, redox homeostasis of the IMS needs to be maintained in spite of its proximity to the ETC. The ETC contains major sites of ROS production, which were traditionally envisioned as merely harmful molecules contributing to aging and disease. It is now becoming clearer that an exquisite balance between ROS production and scavenging is essential to maintain mitochondrial activity. Another emerging concept is that they constitute signaling molecules important for a correct mitochondrial biogenesis, mediating feedback mechanisms regulating the biogenesis and degradation of ETC components. The mechanisms and players underlying these phenomena are just starting to be unraveled. In this respect, an interesting hypothesis to explore is the fact that ROS might be a sensor of unbalanced ETC activity, triggering mechanisms aimed to compensate it, through biogenetical factors and proteases whose activity is directly redox regulated.

## Author Contributions

SG and EF-V wrote the first draft and produced the figures and table. EF-V and KT coordinated the writing. KT obtained funding. All authors edited the final version.

## Conflict of Interest

The authors declare that the research was conducted in the absence of any commercial or financial relationships that could be construed as a potential conflict of interest.

## Publisher’s Note

All claims expressed in this article are solely those of the authors and do not necessarily represent those of their affiliated organizations, or those of the publisher, the editors and the reviewers. Any product that may be evaluated in this article, or claim that may be made by its manufacturer, is not guaranteed or endorsed by the publisher.
